# Clinical Risk Factors Associated with Anti-Epileptic Drug Responsiveness in Canine Epilepsy

**DOI:** 10.1371/journal.pone.0106026

**Published:** 2014-08-25

**Authors:** Rowena M. A. Packer, Nadia K. Shihab, Bruno B. J. Torres, Holger A. Volk

**Affiliations:** 1 Department of Clinical Science and Services, Royal Veterinary College, Hatfield, Hertfordshire, United Kingdom; 2 Department of Neurology/Neurosurgery, Southern Counties Veterinary Specialists, Ringwood, Hampshire, United Kingdom; 3 Department of Veterinary Medicine and Surgery, Federal University of Minas Gerais, Belo Horizonte, Minas Gerais, Brazil; University of Modena and Reggio Emilia, Italy

## Abstract

The nature and occurrence of remission, and conversely, pharmacoresistance following epilepsy treatment is still not fully understood in human or veterinary medicine. As such, predicting which patients will have good or poor treatment outcomes is imprecise, impeding patient management. In the present study, we use a naturally occurring animal model of pharmacoresistant epilepsy to investigate clinical risk factors associated with treatment outcome. Dogs with idiopathic epilepsy, for which no underlying cause was identified, were treated at a canine epilepsy clinic and monitored following discharge from a small animal referral hospital. Clinical data was gained via standardised owner questionnaires and longitudinal follow up data was gained via telephone interview with the dogs’ owners. At follow up, 14% of treated dogs were in seizure-free remission. Dogs that did not achieve remission were more likely to be male, and to have previously experienced cluster seizures. Seizure frequency or the total number of seizures prior to treatment were not significant predictors of pharmacoresistance, demonstrating that seizure density, that is, the temporal pattern of seizure activity, is a more influential predictor of pharmacoresistance. These results are in line with clinical studies of human epilepsy, and experimental rodent models of epilepsy, that patients experiencing episodes of high seizure density (cluster seizures), not just a high seizure frequency pre-treatment, are at an increased risk of drug-refractoriness. These data provide further evidence that the dog could be a useful naturally occurring epilepsy model in the study of pharmacoresistant epilepsy.

## Introduction

Epilepsy is the most common chronic neurological condition in humans and dogs, with estimated prevalences of 0.4–1% [1] and 0.6%, respectively [2]. In human medicine, the best improvement in Quality of Life (QoL) for epilepsy patients is achieved when treatment leads to remission (seizure freedom) [3]–[5]. Indeed, in one study, no significant change in QoL was found after treatment for subjects that did *not* achieve seizure freedom [4]. In addition to anti-epileptic drug (AED) therapy, surgical interventions are utilised to achieve seizure freedom in medically intractable cases [6]. The dog has been considered as a naturally occurring model of human epilepsy [7], [8]. There are considerable parallels in the diagnosis of human and canine epilepsy, with similarly high levels of workup, for example and the use of advanced diagnostic imaging and in limited cases, the use of electroencephalography (EEG) [9]. However, in veterinary medicine, most epilepsy trials have primarily focused on reducing seizure frequency, rather than achieving seizure freedom. Indeed, an ≥50% reduction in seizure frequency has been the definition of AED efficacy in the majority of canine epilepsy studies (e.g. [10]–[17]). This may not be a satisfactory outcome for the carers (the owners), with nearly one third considering only complete seizure freedom as an acceptable outcome [18]. More than two thirds of dogs with epilepsy will continue to have seizures long-term [19]–[22] and around 20–30% will remain poorly controlled (<50% reduction of seizure frequency) despite adequate treatment with phenobarbitone (PB) and/or potassium bromide (KBr) [23]–[25]. Consequently, there is a need to identify those dogs that are likely to have poor outcomes so that owners have realistic, evidence-based expectations of their dog’s treatment. This has been an area of focus in human epilepsy, with analyses identifying risk factors for pharmacoresistance and poor outcome (e.g. [26]–[28]). In contrast, it has been recognised that more epidemiologic studies are needed to further document the nature and occurrence of remission of epilepsy in dogs [29], and identify risk factors associated with positive and negative outcomes. For those dogs that are unresponsive to AEDs, ‘alternative’ non-pharmacological treatment options need to be developed to improve their quantity and quality of life, for example, dietary and surgical interventions [30].

Remission with or without medication has been observed in canine epilepsy cases, demonstrating that epilepsy in dogs is not *necessarily* a lifelong condition. Remission rates vary between studies, for example in a study of Danish Labrador Retrievers, 24% of dogs were classed as being in remission; with only 1 (6%) of these receiving antiepileptic treatment (drug-induced remission) [21]. In a further Danish study of 63 dogs with epilepsy, the remission rate (both spontaneous remission and remission with treatment) was 15% [22]. In these studies, remission was classified as being seizure free for two years or three years seizure free, respectively. In a Swiss study of Labrador Retrievers, 30% of dogs treated with phenobarbitone became seizure-free, with an average follow-up period of 4.8 years [19]. In a study of the efficacy of phenobarbital compared with KBr as a first line treatment, complete seizure freedom was achieved in 85% and 52%, respectively, of treated dogs [31]. This study only lasted for six months however, and it is possible that the percentage of dogs experiencing seizure freedom would be lower given a longer follow-up period. In addition, higher % treatment success rates may reflect studying animals in first opinion practice environment, where seizure phenotypes are likely to be less severe than animals seen at referral practices.

Several factors related to the natural history of the disease and clinical factors have been implicated in both the experimental and clinical literature as influencing the likelihood of successful treatment with AEDs (either remission or <50% reduction in seizure frequency). For example, recent rodent studies found that early treatment [32] had a positive influence on the likelihood of remission being achieved in certain types of epilepsy. Indeed, in human epilepsy it was thought that patients should be treated with AEDs immediately after a seizure to increase the likelihood of achieving remission. However, evidence that remission rates in countries with and without ready access to AEDs are similar [33] implies that AEDs may act to suppress seizures, but have no influence on achieving remission. In addition, there is increasing evidence from both canine, rodent and human studies, that other aspects of disease e.g. different markers of severity *can* influence drug responsiveness and treatment outcome [19], [29], [34]–[36]. This includes a high seizure frequency before treatment, and the presence of cluster seizures and/or status epilepticus. Much of the canine epilepsy literature in this area is derived from single breed studies, thus the aim of this retrospective study was to investigate factors associated with remission in a large population of dogs with epilepsy treated at a multi-breed canine specific epilepsy clinic.

## Materials and Methods

Data from dogs treated at a multi-breed canine specific epilepsy clinic at the Royal Veterinary College Small Animal Referral Hospital (RVC SARH) between 2005–2011 was retrospectively collected from RVC’s electronic patient records. Clinical data was originally gained via standardised owner questionnaires for epilepsy patients at their first appointment, and longitudinal follow up data was gained via telephone interview with the dogs’ owners. All dogs received a uniform diagnostic protocol (including complete blood cell count; serum biochemical profile and dynamic bile acid testing; MRI of the brain, 1.5-Tesla Gyroscan NT, Philips Medical Systems) and a neurological examination to rule out an underlying cause of the seizure activity. Only dogs which were reported in the records to be diagnosed with idiopathic epilepsy, for which a cause was not identified (no remarkable findings on interictal neurological examination, haematology, biochemistry, brain magnetic resonance imaging and cerebrospinal fluid examination), were included in the study. A genetic or hereditary basis cannot be confirmed for every case included in the study, and it is possible that the cause could have been identified with continuous EEG recording. Only dogs receiving AEDs were included in the study.

Seizures were classified according to the former guidelines of the International League Against Epilepsy, modified for veterinary patients (Berendt and Gram, 1999; Licht et al., 2002). Epilepsy was defined of at least two unprovoked seizures >24 h apart. Cluster seizures were defined as an episode where more than one seizure occurred within a 24 h period, with full recovery of consciousness between seizures. Status epilepticus was defined as seizure activity lasting longer than 10 min without gaining consciousness. Seizure activity lasting less than 10 min without gaining consciousness was classed as a single seizure episode. A consistent history was collected with the help of a questionnaire developed for a previous study [10]. The data collected included: signalment, age presented to the hospital (days), age of dog at the time of the first seizure (days), time until diagnosis (days), duration of the disorder before treatment (days), number of seizures prior to any treatment with an AED, seizure frequency per month before medication, type of seizures experienced, and experience of cluster seizures (yes/no) and status epilepticus (yes/no). Medication administered was recorded, specifically whether phenobarbitone (PB), potassium bromide (KBr) or other 3^rd^ line drugs were prescribed, and response to these drugs recorded as responsive or unresponsive. Follow up time was recorded in days. Treatment success was recorded as:

- Seizure-free remission (with or without medication) (1/0)
- ≥50% reduction in seizure frequency (1/0)

Non-responsiveness to an AED was classified as a less than 50% reduction in seizure frequency, despite being within the reference range for the prescribed AED(s) and titrated to the maximum tolerated effective dose. As these data were derived from a clinical population, decision-making leading to the maximum dose of any AED was made by both the clinician and the owner, taking into account adverse effects of the drug and its efficacy. Serum levels of phenobarbitone and/or potassium bromide were checked by the attending clinician, and recorded from the clinical records where available, to ensure the dog was within the reference range for these AEDs and receiving adequate therapy, and to test the effect of this variable.

### Ethics statement

This study was approved by the Royal Veterinary College’s Ethics and Welfare Committee. The owners of the dogs gave permission for their animals to be used in this study.

### Statistical analysis

Differences between outcome variables were tested with a Fisher’s exact test for categorical variables with expected values <10, and the Pearson’s chi squared test for expected values >10. The Mann-Whitney U-test was used for continuous variables. Generalised linear mixed models for binary outcomes were then used to identify risk factors in a multivariate analysis for successful treatment outcomes, using the lmer function in R from the lme4 package. Treatment outcomes (i) seizure free remission with or without medication (1/0) and (ii) ≥50% reduction in seizure frequency (1/0) were used as the response variables in models. Follow-up time and serum AED values were tested in the models to verify that they did not have an effect on treatment success. Breed was included as a random effect, with all cross breeds coded plainly as ‘cross breed’ due to the unknown parentage of many of these dogs. This random effect took into account the genetic non-independence of multiple members of the same breed in the study population, and possible demographic and environmental factors. Predictors including age, sex and neuter status were tested in all models. Multicollinearity was checked for in all models, identified from inflated standard errors in the models, and thus avoided. Model fit was assessed using the deviance and Akaike’s information criterion. Data is presented as median with 25^th^ and 75^th^ percentiles and all tests were used two-sided with *P*<0.05 being considered statistically significant.

## Results

### Population demographics

122 dogs were lost to follow and 344 dogs were included in the analysis, of which 89.5% were pure bred and 10.5% were cross-breeds. The five most common breeds were the Labrador Retriever (14.8%), cross breed (10.5%), Border Collie (9.9%), German Shepherd Dog (8.7%) and the Staffordshire Bull Terrier (5.5%). The majority of dogs were male (70.3%), with 57% of all dogs neutered. The median age (in days) at presentation to the small animal referral hospital was 1260 days (720–2008) (approximately 3.5 years).

### Clinical data

The median age at onset of seizures was 780 days (360–1447.5). The median time until diagnosis was 150 days (38–360), with the median duration of the disorder before treatment 67.5 days (30–180). The median number of seizures before the start of treatment was 4.5 (3–7.25) with a median seizure frequency (per month) before medication of 3 (1–5). The median follow up time was 656 days (330–960).

A minority of dogs had experienced status epilepticus (13.1%), whereas nearly half of dogs had experienced cluster seizures (48%). There was a significant association between the presence of status epilepticus and cluster seizures (X^2^ = 8.05, P = 0.004), with 9.8% of dogs experiencing both status epilepticus and cluster seizures. There was no difference between male and female dogs experiencing cluster seizures (48.9% *vs*. 45.8%; X^2^ = 0.26, P = 0.61); however, more male dogs experienced status epilepticus than female dogs (15.5% vs. 5.2%; X^2^ = 4.12, P = 0.041). At the univariate level (Table 1) dogs without cluster seizures were significantly more likely to go into remission, but there was no difference in dogs with or without status epilepticus.

**Table 1:** Association between clinical variables and being in seizure-free remission in canine epilepsy patients.

		Remission		Statistics	
		No (%)	Yes (%)	Fishers exact (2 sided)	*P*
Sex	Male	75.1	53.6	5.56	0.024
	Female	24.9	46.4		
Neuter status	Neutered	53.2	75.0	4.53	0.038
	Entire	46.8	25.0		
Seizure severity	Statusepilepticus	20.0	0.0	0.25	0.802
	No Statusepilepticus	80.0	100.0		
	Clusterseizures	62.8	17.9	19.63	<0.001
	No Clusterseizures	37.2	82.1		
		**Median (25^th^–75^th^ percentile)**	**Median (25^th^–75^th^ percentile)**	**Mann Whitney U**	***P***
Age presented to hospital(days)		1080 (720–1800)	1440 (1080–2085)	1933	0.61
Time until diagnosis (days)		180 (62.3–378.8)	90 (15–225)	1204	0.79
Age at onset seizures (days)		720 (441–1286)	1170 (720–1725)	2971	0.026
Duration of disorder beforetreatment (days)		90 (30–180)	60 (26–120)	578	0.31
Number of seizures beforestart of treatment		5 (3–8.5)	4 (3–5.3)	1286	0.09
Seizure frequency per monthbefore medication		3 (1–6)	2 (1.25–3.75)	1582	0.39

The most common seizure type was complex-focal seizures with secondary tonic-clonic generalisation (35.7%), followed by generalised tonic-clonic (32.6%), complex-focal (14.1%), and simple-focal seizures with secondary tonic-clonic generalisation (13.7%). The rarest seizure type was simple-focal seizures with only 11 cases (3.8%).

Of the 113 dogs for which PB concentrations were available, they were well within the reference range (29.1±1.60 µg/ml, reference range from our laboratory of 15–45 µg/ml). KBr concentrations were available for 53 dogs and were 1.61±0.11 mg/ml, again well within the reference range from our laboratory of 0.5–1.9 mg/ml.

The majority of dogs were receiving PB at follow up (67.2%), with a further 38.4% of cases receiving KBr, and 27% of all cases receiving PB and KBr in combination. A minority of cases (10.2%) were prescribed a third line AED (e.g. gabapentin, pregabalin, levetiracetam and zonisamide). In addition, 5.4% of cases received emergency rectal diazepam treatment and 8.1% received pulsed intermittent treatment with levetiracetam.

### Risk factors for remission

Fourteen per cent of dogs were in remission on PB treatment. When ≥50% reduction in seizure frequency is used as the outcome measure, success rates are markedly higher with 64.5% of dogs achieving this level of seizure reduction. At the univariate level, several factors were associated with an increased likelihood of achieving remission (Table 1), namely: being female, neutered, no previous experience of cluster seizures and an older age at onset of seizures. The same four factors were also associated with an increased likelihood of achieving an ≥50% reduction in seizure frequency, with the addition of an older age at presentation to hospital (Table 2).

**Table 2:** Association between clinical variables and ≥50% reduction in seizure frequency in canine epilepsy patients.

		≥50% reduction		Statistics	
		No (%)	Yes (%)	Fishers exact (2 sided)	*P*
Sex	Male	78.5	64.5	5.54	0.025
	Female	21.5	35.5		
Neuter status	Neutered	50.0	63.2	3.62	0.040
	Entire	50.0	36.8		
Seizure severity	Statusepilepticus	21.1	10.2	4.35	0.052
	No Statusepilepticus	78.9	89.8		
	Clusterseizures	71.7	33.5	34.01	<0.001
	No Clusterseizures	28.3	66.5		
		**Median (25^th^–75^th^ percentile)**	**Median (25^th^–75^th^ percentile)**	**Mann Whitney U**	***P***
Age presented to hospital(days)		990 (720–1514.8)	1424.5 (840–2094.5)	5795	0.011
Time until diagnosis (days)		183 (72.5–360)	150 (34–360)	4225.5	0.216
Age at onset seizures (days)		720 (360–1125)	968 (447.8–1699)	9893	0.007
Duration of disorder beforetreatment (days)		37.5 (22.5–142.5)	90 (30–180)	833.5	0.064
Number of seizures beforestart of treatment		5 (3.3–8.8)	4.5 (3–7.8)	2762	0.276
Seizure frequency permonth before medication		3 (1–5)	2 (1–5)	5022.5	0.569

When tested in a multivariate mixed model (Table 3), two categorical variables were significantly associated with the likelihood of remission being achieved; sex and cluster seizures, with female dogs over two times more likely to achieve remission, and dogs with no previous experience of cluster seizures over six times more likely to achieve remission. No effects of neuter status or previous episodes of status epilepticus were found in any model, and were not found to improve model fit (determined by Akaike Information Criterion [AIC] and % correct classification), and as such they were not included in the final model. There were no significant effects of time until diagnosis, duration of time before treatment, the number of seizures before treatment or the seizure frequency per month before medication. No effects of follow up time or serum AED values were found. There were no significant effects of seizure type on the likelihood of remission (p = 0.208); however the seizure types with the lowest remission rates were simple-focal (0% remission) and complex-focal seizure with secondary tonic-clonic generalisation (14.1% remission).

**Table 3:** Risk factors for remission in canine epilepsy cases.

Predictor	Odds Ratio (95% CI OR)		SE (coef)	Z	*P*
Sex					
Female	2.39 (1.01–5.64)		0.44	2.00	0.047
Male	*Ref*				
Cluster Seizures					
No	6.08 (2.35–15.70)		0.49	3.75	<0.001
Yes	*ref*				

When an ≥50% reduction in seizure frequency is used as the outcome measure (Table 2 and 4), the same two factors were found to significantly predict the likelihood of achieving remission in a multivariate model (Table 4), with the addition of age at onset of seizures. As age at onset of seizures increases, the likelihood of achieving an ≥50% reduction in seizure frequency increases.

**Table 4:** Risk factors for an ≥50% reduction in seizure frequency in canine epilepsy cases.

Predictor	Odds Ratio (95% CI OR)	SE (coef)	Z	*P*
Sex				
Female	2.15 (1.12–4.15)	0.33	2.32	0.021
Male	*ref*			
Cluster Seizures				
No	4.66 (2.58–8.39)	0.30	5.14	<0.001
Yes	*ref*			
Age at onset of seizures (days)	1.00 (1.00–1.01)	0.00	2.51	0.013

### Breeds

Dogs of fifteen different breeds achieved seizure freedom, and dogs of fifty-two breeds achieved an ≥50% reduction in seizure frequency. There was no statistically significant effect of breed on the likelihood of dogs going into remission or having an ≥50% reduction in seizure frequency when tested at the univariate level. Of the breeds with over 10 dogs for which data was available (the Labrador Retriever, Cross Breed, German Shepherd, Border Collie and Staffordshire Bull Terrier), the breed least likely to go into remission or have an ≥50% reduction in seizure frequency was the Border Collie (0% and 40% respectively), followed by the German Shepherd (11% and 35%) and Staffordshire Bull Terrier (0% and 57%). Fishers exact tests revealed only significant effects of being a Border Collie or German Shepherd on the likelihood of entering remission or experiencing an ≥50% reduction in seizure frequency (Table 5). When these breeds were included in multivariate analyses as binary variables, no significant effects were found.

**Table 5:** Top five breeds most likely to lack drug response.

Breed	% remission	*p*	% ≥50% reduction	*P*
Border Collie	0	0.02	40	0.01
German Shepherd	11	0.51	35	0.01
Staffordshire Bull Terrier	0	0.18	57	0.37
Cross Breed	19	0.30	61	0.38
Labrador Retriever	23	0.14	76	0.07

## Discussion

The results of this retrospective study provide evidence that the presence of cluster seizures and thus seizure *density* (the temporal pattern of seizure activity) is a more influential risk factor on the likelihood of achieving remission in canine epilepsy than seizure frequency or the total number of seizures prior to treatment. Nearly half (48%) of dogs in the study population had experienced cluster seizures, of which only 17.9% achieved remission and 33.5% achieved an ≥50% reduction in seizure frequency. This result has previously been found in human epilepsy [37]. The number of epileptic dogs that experience cluster seizures varies between studies, with recent reports between 38% and 64% [20], [38]. The breed least likely to achieve remission in this study was the Border Collie, a breed previously demonstrated to have a higher level of cluster seizures than other breeds (84.6% affected) [20], with similar levels reported in other studies (e.g. 94%; [29]). A remission rate of 14.2% was observed in this study, similar to a previous Danish study of canine epilepsy (15%) [22]. These were both mixed study populations; however, in studies of Labrador Retrievers in isolation, higher levels of remission have been observed (24–40%) [19], [21]. When >50% reduction in seizure frequency is used as the outcome measure, success rates are markedly higher at 64.5%.

Seizure *density* as well as frequency has been demonstrated to influence the likelihood of remission in humans, with individuals who experience an episode of status epilepticus [39]–[41], or cluster seizures [37] less likely to go into remission. These results were also seen in a recent study of predictors of pharmacoresistance in rats, where the average seizure frequency per day of 13 rats nonresponsive to medication was 4.31/day, indicating some rats having cluster seizures [36]. This frequency was significantly higher than 20 drug-responsive rats (mean 0.54/day). It is further notable, that of the 13 rats that were unresponsive to medication, a subgroup of six rats (18%) experienced high levels of cluster seizures, with an average of 8.94 seizures per day [36]. Intact male and female dogs have a higher likelihood of having cluster seizures [42] which may have a negative impact on their prognosis. Evidence from canine epilepsy is not clear however, with 89% (8/9) of Border Collies in remission having a history of cluster seizures, status epilepticus, or both [29]. A severe epilepsy phenotype is often seen in this breed, thus data from a larger population with a diversity of breeds represented would be valuable to gain an insight into this relationship in a wider population with a variety of disease phenotypes.

No evidence was found to support the results of a recent rodent study that found early treatment [32] influenced the likelihood of remission being achieved. There are divergent opinions within the veterinary profession regarding time to treatment after diagnosis of epilepsy, a topic also debated in human medicine [43]. One school of thought advises treatment of seizures as soon as a dog is diagnosed as having recurrent seizures (i.e. after the second seizure episode). However, the impact of AED side effects on QoL may be considerable, with this being the top reason cited by owners for a decreased QoL in their dogs (28% of 25 owners questioned) [44]. As such, the second school of thought considers that there should be a balance between the benefits gained from using AEDs with the potential adverse effects they cause. The results of this study indicated no effect of time to treatment; however, there is mixed evidence regarding its effects on treatment outcome. In clinical studies of epilepsy in dogs, decreased time to treatment has not been observed as a positive influence upon treatment outcome, indeed, one study demonstrated that Labrador Retrievers that were in remission received medication a longer period of time after their first seizure than those dogs which continued to seizure [19]. It should be acknowledged that this result may be biased by animals with a more severe seizure phenotype receiving treatment earlier, due to owner and/or veterinarian concerns. It is currently not veterinary practice to initiate treatment after the first seizure. Early initation of treatment has also proven unsuccessful in several human studies [45]–[47]. Time to treatment is additionally likely to be influenced by disease severity, for example it was shorter in dogs with episodes of status epilepticus [48], thus being confounding factors in statistical analyses.

A large number of seizures before treatment has been identified as a poor prognostic factor in several previous human studies of epilepsy [34], [41], [49], with patients experiencing a greater number of seizures prior to initiation of treatment more likely to have refractory epilepsy. In rats, it was recently demonstrated that seizure frequency in the early phase of epilepsy is a strong predictor of refractoriness [36]. This has also been seen in dogs, with refractory dogs having a significantly higher number of seizures prior to presentation and beginning of treatment in Labradors [19] and an initially higher seizure frequency in Border Collies [29]. It has been discussed whether this initial high seizure frequency and subsequent refractoriness may be an effect of kindling (Reynolds, 1995). However, as time to treatment has not been found to be a strong predictor of refractoriness in dogs and humans, initial high seizure frequency has been considered more likely to be the result, rather than the cause of the pathophysiological changes that are later manifested as refractory epilepsy [34], [50]. Indeed, in this study and another previous study of canine epilepsy, the number of seizures before treatment was not significantly different between dogs positive *vs*. negative treatment outcomes [48]. In addition, no effect was found of seizure type upon the likelihood of remission; however, the most common seizure type in dogs that did not achieve remission (39.6%) was complex-focal seizures, also seen in human epilepsy [51], [52], adding evidence to the belief that focal seizures are more challenging to treat.

Males were found to be less likely to achieve remission than female dogs. Historically, male dogs are thought to seizure more than female dogs [53], and recent epidemiological studies of idiopathic epilepsy have confirmed a male overrepresentation for this disorder [2], [38]. With regard to the impact of sex upon treatment outcome, little existing data is available. One study noted that female dogs with epilepsy lived longer with the disorder than male dogs, with a median age at death two years greater (8 vs. 6 years, respectively) [22]; however, this outcome measure may be influenced by owner euthanasia decisions, so can only be a proxy of treatment success. In previous studies, male dogs were found to be more highly affected by cluster seizures than female dogs [42]. This result was not found in the current study, and indeed sex and the presence of cluster seizures were found to be independently significant risk factors, thus further investigation is warranted into the effect of sex on treatment outcome.

Age at onset of disease was found to significantly influence the likelihood of achieving an ≥50% reduction in seizure frequency, with dogs experiencing their first seizure at an older age more likely to achieve this level of reduction. This has previously been demonstrated in Border Collies, with the mean age at onset significantly higher in dogs with remission compared to those with active epilepsy [29], and in Labradors, with dogs classed as having excellent or good results (defined as those that were seizure-free, or had an improvement in their seizure frequency, strength and/or duration) having a significantly higher age at onset than uncontrolled dogs [19]. Early age at seizure onset has been previously identified in children to be a predictor of pharmacoresistance [54]. In contrast, in a study of canine juvenile epilepsy (where the first seizure occurs before the age of one year), age at onset had no influence on survival outcome [20].

There are recognised limitations to studying epilepsy in a veterinary referral population [20] due to a bias towards a more severe seizure phenotype, and thus may not be representative of the whole canine epilepsy population. As such, further studies of epilepsy in the first opinion practice population may be warranted, although the level of diagnostic work up may be lower owing to availability of equipment and specialist expertise, and thus confidence in diagnosis may be variable. A further limitation of this study is the varied follow up time of cases. In previous studies, remission was strictly classified as dogs that were seizure free for two or three years [21], [22]. In human epilepsy, seizures may re-occur after a period of months of seizure freedom, without alterations to treatment [55]. As such, some of the dogs classified as seizure free in this study may have later experienced seizures. This may be due to a variety of factors including drug tolerance, deterioration of the epilepsy phenotype, acquired drug resistance and poor owner compliance [55]. The median follow up time, however, was 656 days and thus is in line with the follow-up standards of comparable epilepsy studies in the veterinary environment. There are limitations to which variables could be controlled for in this study, introduced by data being collected in a clinical environment with naturally occurring disease in client owned animals. Due to the expense of medication it is possible that clients may decline third line AEDs that may affect the response rate, which may mean the figures here are an underestimate of how many dogs could biologically achieve remission. In addition, EEG is not routinely used in the Canine Epilepsy Clinic data were sourced from, or in veterinary medicine in general at present, and thus there is no confirmation that the ‘seizure’ episodes reported by owners was indeed seizure activity. A further limitation of this study is that serum AED levels were not available for all dogs; however, clinicians contributing to this dataset routinely checked AED serum levels were within the reference ranges and thus, this is naturally standardised across the sample. Furthermore, not all serum levels were conducted at the same laboratory and therefore could not be analysed in the same dataset. No statistical association between AED levels and treatment outcome were found; however, future studies could include this variable to check this result was not due to the lower power of this sub-sample.

## Conclusions

In conclusion, the present provides evidence that it is not merely the absolute *number* of seizures prior to treatment that predicts refractoriness, but their temporal pattern, with those patients experiencing cluster seizures (more than one seizure within a 24 h period) more likely to be pharmacoresistant. Whether this result is an effect of cluster seizures promoting epileptogenesis and causing brain damage that results in seizures resistant to medication, or is actually a reflection of a more aggressive disease phenotype that is harder to treat is unknown, and warrants further investigation. The present study further demonstrates similarities between this naturally occurring model of epilepsy and both experimental rodent models [36], and human clinical studies [37]. The similarity between clinical environments, high level of diagnostic work up, and shared living environments between humans and dogs further strengthens the use of this readily available animal model.
